# Maternal high fat diet compromises survival and modulates lung development of offspring, and impairs lung function of dams (female mice)

**DOI:** 10.1186/s12931-019-0976-3

**Published:** 2019-01-30

**Authors:** Jordan Smoothy, Alexander N. Larcombe, Emily K. Chivers, Vance B. Matthews, Shelley Gorman

**Affiliations:** 1 Telethon Kids Institute, University of Western Australia, Northern Entrance Perth Children’s Hospital, 15 Hospital Ave, Nedlands, Western Australia 6009 Australia; 2 School of Biomedical Sciences, University of Western Australia, Perth, Australia; 3 School of Public Health, Curtin University, Perth, Western Australia 6845 Australia

**Keywords:** Maternal obesity, Lung function, Lung development, Inflammation, Immune training

## Abstract

**Background:**

Epidemiological studies have identified strong relationships between maternal obesity and offspring respiratory dysfunction; however, the causal direction is not known. We tested whether maternal obesity alters respiratory function of offspring in early life.

**Methods:**

Female C57Bl/6 J mice were fed a high or low fat diet prior to and during two rounds of mating and resulting pregnancies with offspring lung function assessed at 2 weeks of age. The lung function of dams was measured at 33 weeks of age.

**Results:**

A high fat diet caused significant weight gain prior to conception with dams exhibiting elevated fasting glucose, and glucose intolerance. The number of surviving litters was significantly less for dams fed a high fat diet, and surviving offspring weighed more, were longer and had larger lung volumes than those born to dams fed a low fat diet. The larger lung volumes significantly correlated in a linear fashion with body length. Pups born from the second pregnancy had reduced tissue elastance compared to pups born from the first pregnancy, regardless of the dam’s diet. As there was reduced offspring survival born to dams fed a high fat diet, the statistical power of lung function measures of offspring was limited. There were signs of increased inflammation in the bronchoalveolar lavage fluid of dams (but not offspring) fed a high fat diet, with more tumour necrosis factor-α, interleukin(IL)-5, IL-33 and leptin detected. Dams that were fed a high fat diet and became pregnant twice had reduced fasting glucose immediately prior to the second mating, and lower levels of IL-33 and leptin in bronchoalveolar lavage fluid.

**Conclusions:**

While maternal high fat diet compromised litter survival, it also promoted somatic and lung growth (increased lung volume) in the offspring. Further studies are required to examine downstream effects of this enhanced lung volume on respiratory function in disease settings.

## Background

Obesity increases the risk of asthma development by > 3-fold [1]. There are many plausible explanations for how obesity could cause asthma. These include environmental influences, genetic predisposition, comorbidities, mechanical changes associated with increased body weight, direct contribution of adipokines like leptin, and low-grade systemic inflammation induced by obesity. There is also the possibility that foetal programming events that occur via maternal obesity could contribute towards asthma development in childhood. Most epidemiological analyses have concluded that maternal pre-pregnancy obesity is linked with an increased risk of wheezing in the first years of life and childhood asthma (reviewed by [2]). These observations around increased risk for wheeze in early life are likely to be independent of maternal atopy or asthma [2]. There is up to a 50% increased odds of asthma in children of obese/overweight mothers (reviewed in [3]). These observations suggest that maternal obesity could contribute significantly towards asthma susceptibility and other associated respiratory problems (like those induced by viral infection).

The in utero and neonatal periods are particularly critical as abnormal weight gain at these times may have irreversible or exacerbating effects on the development of both obesity [1] and asthma [4]. Maternal obesity may induce foetal programming of the immune, metabolic, and respiratory systems to increase the chance of childhood asthma, but maternal obesity is a complex exposure, which could be related to a range of dietary, environmental and genetic factors. While direct foetal programming could explain the observed positive relationships between maternal obesity and offspring asthma, it is quite plausible that they are confounded by either shared environmental or genetic risk factors for both maternal obesity and childhood asthma. Epidemiological studies do not prove that maternal obesity is a cause of childhood asthma, and may be confounded by environmental or genetic risk factors, for example, excessive gestational weight gain [5].

Furthermore, the mechanism(s) by which maternal obesity increases asthma risk in offspring are yet to be determined. Some suggested biological mechanisms for the maternal obesity-childhood asthma link include: foetal exposure to high concentrations of proinflammatory cytokines and adipokines, stress hormones, oxidative stress and glucose [5, 6]; placental inflammation and dysfunction [2]; increased rates of pregnancy, delivery and neonatal complications [2, 7]; increased use of reproductive technologies and prescriptive medications [2]; and, an involvement of intestinal [2, 7] or lung microbiota [8]. Given the complexity of maternal obesity as an exposure, animal models promise to help elucidate the nature and mechanisms behind this association.

The ability of maternal obesity to promote the development of obesity in offspring is well established in murine models [9]. High fat diet intake by murine dams during pregnancy and lactation induces a phenotype in offspring that closely resembles human metabolic syndrome with abnormal glucose homeostasis, increased blood pressure, abnormal serum lipid profiles, increased adiposity and insulin resistance [9]. Feeding mice a high fat diet can also induce inflammation, whereby increased eosinophils, interleukin-5 (IL-5), eotaxin, tumour necrosis factor (TNF)α and leptin were observed in the lungs with the induction of allergic airway disease [10]. Other studies report similar effects of a high fat diet to induce lung eosinophilia, but without adverse effects on lung function [11]. However, not all studies report adverse effects of consuming a high fat diet in mice with allergic airway disease [12].

More recent studies have identified that maternal high fat diet or over-nutrition reduces surfactant mRNA or protein expression in the lungs of foetal and neonatal mice [13–15], with some researchers identifying adverse effects on lung development [13], with effects on lung function in early life not yet described. These are important studies to complete, and may help us determine the potential of maternal obesity and metabolic dysfunction, and high fat diet exposure in early life to contribute towards the development of asthma in childhood. We hypothesized that maternal obesity and metabolic dysfunction (induced pre-conception) would modify the lung function of offspring in early life to potentially contribute towards the development of respiratory dysfunction in later life. To test this, we used a model of maternal high fat intake to induce substantial weight gain and metabolic dysfunction prior to mating, with a high fat diet fed to mice throughout pregnancy and lactation periods. We then tested the respiratory (and other health outcomes) of 2 week-old offspring (before weaning), and dams, using our optimised forced oscillation technique (FOT) in which we measured lung function in neonatal mice at 2 weeks of age [16–19], the earliest age (and size) for which this is currently technically possible in mice.

## Methods

### Mice

Experiments were performed according to the ethical guidelines of the National Health and Medical Research Council of Australia and with approval from the Telethon Kids Institute Animal Ethics Committee (AEC#280). C57Bl/6 J male and female mice were purchased from the Animal Resources Centre, Western Australia and maintained under specific-pathogen-free conditions. C57Bl/6 J mice were chosen due to their increased susceptibility for developing signs of obesity and insulin resistance when fed a high fat diet compared with other mouse strains [20, 21]. Female mice (dams-to-be) were randomly allocated into the low fat diet and high fat diet treatment groups. Experiments were performed in animals from January to October 2015. All mice were caged in open-topped cages initially in pairs until pregnancies were confirmed (and then housed individually) under 12 h:12 h standard light:dark conditions at 23.0 ± 1.0 °C (mean ± SD). A female mouse fed a high fat diet was euthanized when it experienced a seizure after consuming the high fat diet for 28 weeks, before the final lung function measurements could be obtained.

### Diets

Female mice were fed a low fat (SF12–030, Specialty Feeds, Perth, Western Australia) or high fat diet (SF12–032, Specialty Feeds) as previously described [22], with the contents of these diets shown in Table 1. The low and high fat diets had fat contents of 5.0% (canola oil) and 23.5% (20.7% lard and 2.9% canola oil), respectively. The low fat diet had a similar fat content to standard rat/mouse wheat-based diets (those used in our facility have fat contents of 6.0%). Male mice used for breeding purposes were fed a standard rat/mouse wheat-based diet except when housed with females for breeding.

**Table 1:** Composition of the high fat diet (HFD) and low fat diet (LFD) fed to mice

Ingredients (g/100 g)	HFD	LFD
Sucrose	10.0	10.0
Casein (acid)	20.1	20.1
Canola oil	2.9	5.0
Lard	20.7	0.0
Cellulose	5.00	5.00
Wheat starch	21.4	40.2
Dextrinised starch	12.2	13.2
Di methionine	0.30	0.30
Manganese oxide	0.01	0.01
AIN93_trace minerals	0.14	0.14
Lime (calcium carbonate)	1.50	1.50
Salt (Fine sodium chloride)	0.26	0.26
Potassium dihydrogen phosphate	0.42	0.42
Potassium sulphate	0.27	0.27
Potassium citrate	0.25	0.25
Magnesium oxide	0.24	0.24
Dicalcium pohosphate	2.16	1.96
AIN93_Vitamins^a^	1.00	1.00
Choline chloride 75% *w*/w	0.25	0.25
Food Colour	0.007	0.008
Ferrous Sulphate	0.01	0.01
Vitamin D supplement (28,500 IU/g)	0.008	0.008
% Digestible energy - lipid	45.9%	12.0%
% Digestible energy - protein	17.7%	22.0%

^a^ American Institute of Nutrition (AIN) trace minerals number 93 is a nutritional supplement used in rodent food designed to promote maintenance of health in adult rodents [60]

### Experiment overview

**Fig. 1:**
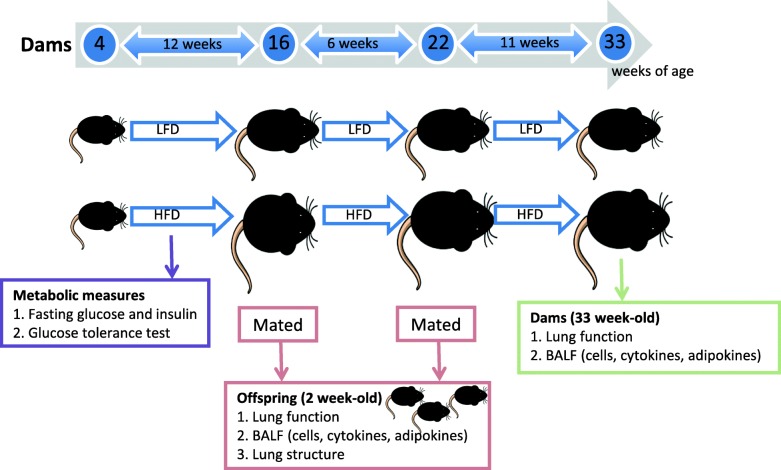
Overview of the experiment*.* From 4 weeks of age, C57Bl/6 J female mice (n = 28) were separated into 2 treatment groups and fed either a low fat diet (LFD, *n* = 14) or high fat diet (HFD, n = 14). Fasting glucose and insulin levels were determined in these female mice after being fed the diets for 5, 7, 9, 11 and 18 (glucose only) weeks. A glucose tolerance test (GTT) was performed after female mice had been fed either diet for 11 weeks. Female mice were first mated with C57Bl/6 J male mice, after dams had been fed either diet for 12 weeks. Three weeks later, offspring were born. Dams were re-mated 1 week after their first litters were removed at 2 weeks of age. The lung function of all surviving offspring (of both pregnancies) was measured when these mice reached 2 weeks of age. After lung function assessments were completed, BALF was collected from most neonates to determine the proportions of major cell types, and levels of cytokines and adipokines. In a small subset of the neonates, lungs were fixed for structural assessment. After eating the LFD or HFD for 29 weeks, the dams had their lung function and responsiveness to methacholine measured, with BALF also collected to determine the proportions of major cell types, and levels of cytokines and adipokines

Four week-old female C57Bl/6 J mice were randomly allocated to 1 of 2 treatment groups, in which they were fed either the low or high fat diet for 29 weeks (see Fig. 1). A number of metabolic measures were recorded for dams throughout the initial 12 weeks of consuming either diet (Fig. 1, and detailed below). After 12 weeks of consuming either diet, female mice were housed with male mice for 14 days as trios (2 females and 1 male) for breeding. Significant weight gain of greater than 4 g within 10 days of co-housing dams with the male mouse was a good predictor of successful impregnation (more so than the presence of vaginal plugs) regardless of whether mice were fed a low or high fat diet. After confirmation of pregnancies, dams were separated and housed individually and visually monitored for the birth of offspring. Dams and their offspring were not disturbed (with no cage changes) from when pregnancies were confirmed and dams were separated into individual cages, until offspring reached 1 week of age, although extra nesting material was provided to dams in the week prior to the birth of their offspring. Mice were mated a second time 1 week after the first litters of 2 week-old mice were removed for acquisition of body weight, metabolic, lung function and other data (Fig. 1). This was done because of the poor survival of litters from the first pregnancies (see Table 3 below), and to examine the potential effect of multiple pregnancies on dam outcomes. Female mice were 22 weeks of age at this time, and had been fed either diet for 18 weeks. Lung function and other respiratory measures were recorded from 2 week-old offspring of the second litters and 33 week-old dams (fed either diet for 29 weeks) (Fig. 1). We chose the 2 weeks of age age time point, as we wanted to examine the effects of maternal high fat diet on lung development and function at the earliest time point currently technically possible [16–19]. By measuring lung function at 2 weeks of age, we can also be reasonably certain that the direct consumption of high fat diet (through food, and not in milk) by offspring is avoided.

### Body weights and weight gain

Dams were weighed mid-morning on Tuesdays using a digital scale (CAS MWP-3000, sensitivity = > 0.01 g, CAS corporation, NJ, USA) with their weights recorded on a weekly basis. The percentage weight gain was calculated by comparing the current weight with the weight of the mouse at 4 weeks of age. Offspring were weighed at 2 weeks of age.

### Fasting glucose and insulin

Mice were fasted for 5 h by placing them in newly made-up cages without food but with access to water [23, 24]. Blood glucose levels were measured in dams at 5, 7, 9, 11 and 18 weeks of being fed either diet by placing a drop of blood (obtained from the tip of the tail) onto a glucose strip (Accu-Chek; Roche, Castle Hill, NSW) into a glucometer (Accu-Chek Performa, Roche) [23, 24]. Fasting insulin levels were measured in serum obtained from blood collected from the tail vein (lateral and dorsal caudal veins) of dams fed the low or high fat diets for 7, 9 and 11 weeks. A rat/mouse insulin ELISA kit (EMD Millipore Corporation, MA, United States of America) was used to measure serum insulin levels according to the manufacturer’s instructions. The limit of detection of this assay was 0.1 ng/mL.

### Glucose tolerance test

After 11 weeks of feeding the female mice either diet, mice were fasted for 5 h and challenged with 1 g glucose/kg via intraperitoneal injection (Phebra, Lane Cove, NSW, Australia). Immediately preceding glucose injection, and at 15, 30, 45, 60, 90 and 120 min post-injection, the tip of the tail was removed and fasting glucose levels recorded using a glucometer (Accu-Chek Performa, Roche) [25, 26].

### Lung volume and lung function in 2 week-old mice

Mice were anaesthetized with an intraperitoneal (i.p.) injection of ketamine (Troy Laboratories, Glendenning, Australia) and xylazine (Sigma-Aldrich) diluted with 0.9% Sodium chloride solution (Baxter Healthcare, Toongabbie, NSW, Australia). Pups were tracheotomised with a 10 mm length of 21 G needle secured with surgical silk before being placed inside a whole-body plethysmograph and connected to a small animal ventilator (Minivent; Harvard, March-Hugstetten, Germany) [16, 27]. Mice were ventilated at 400 breaths/min at a tidal volume of 10 mL/kg and positive end expiratory pressure (PEEP) of 2 cmH_2_O. Once on the ventilator all mice received a slow deep inflation to P_rs_ = 20 cmH_2_O, followed by another inflation 5 min later to standardize the lung volume history. Immediately after this, thoracic gas volume (TGV) was measured by plethysmography. TGV is the volume of gas contained within the chest during plethysmography when the airway opening is occluded. In mice, this is roughly equivalent to the functional residual capacity of the lung. Two electrodes were inserted into the intercostal muscles, the ventilator was stopped and the airway opening and plethysmography box were briefly closed. Six one-second-long box (P_box_) and tracheal (P_trachea_) pressure measurements were recorded using two pressure transducers (Validyne MP45; Validyne Engineering, Northbridge, CA, USA; and model 8507C-2; Endevco, San Juan Capistrano, CA, USA, respectively). Inspiratory breathing efforts were created during the 6 s by stimulating the intercostal muscles with electrical impulses (~ 20 V amplitude and 1–2 s in duration) [28]. The relationship between P_box_ and P_trachea_ was used to calculate TGV by the application of Boyle’s law [16]. The six measurements made for each mouse were averaged such that we present one TGV measurement per individual. After TGV measurement, we used a wave-tube modification of the FOT adapted for small animals to measure respiratory system impedance (Z_rs_) at functional residual capacity [27]. Six Z_rs_ measurements were made per animal, and these were averaged to obtain one value per individual. The constant phase model was used to partition Z_rs_ into Newtonian resistance, tissue damping, tissue elastance and and hysteresivity. Newtonian resistance (R_n_) is the impedance to the flow of air in the non-respiratory portions of the lungs. As the mice were tracheostomised in this study, this refers to the impedance to airflow in the trachea, bronchi, and bronchioles. Tissue damping (G) reflects the energy dissipation in the alveoli. It can also be thought of as the resistance of the respiratory portions of the lungs. Tissue elastance (H) reflects the energy conservation in the alveoli. It can also be thought of as the “stiffness” of the lung parenchyma. Hysteresivity (η; G/H, a measure of the degree of heterogeneity of ventilation) [29]. Inertance values were negligible and are not reported. Due to the high compliance of the chest wall, R_n_ is equivalent to airway resistance (R_aw_) [18]. As reported in the results section, the number of animals per treatment for these outcomes varied based on the mechanical and technical limitations of our lung function assessment equipment, where while we are able to ventilate very small mice and measure Z_**rs**_ using the FOT, when fitting the constant phase model to impedance, if any measurement artefacts occur (e.g. due to cardiac interference, non-optimal placement of the endotracheal tube etc), they can result in a poor model fit and thus render some or all components of the constant phase model fit invalid. In very small mice, as we were studying here, ideal model fits are very difficult to obtain, despite our experience in this area. Further, as we report specific parameters of lung function for 2-week old offspring (which are corrected to TGV), a valid measurement of TGV is required for each experimental animal. We are not able to analyse TGV measurements “on-the-fly” so it is only later that we are able to tell with certainty that any particular animal has a valid TGV measurement. If that animal does not have a useable TGV, it is not possible to report specific parameters of lung function.

### Responsiveness to methacholine in dams

Lung function and responsiveness to methacholine were measured in dams using the legacy Scireq flexiVent (Scireq, Montreal, Canada) [18]. The flexiVent system was required to obtain responsiveness measurements to MCh as the wave-tube system used for the pups does not allow methacholine to be introduced into the ventilatory circuit. As with the pups, dams were anaesthetized with an intraperitoneal (i.p.) injection of ketamine and xylazine (diluted with 0.9% Sodium chloride solution prior to being tracheotomised with a 1 cm length of cannula (internal diameter 0.086 cm). They were then attached to the flexiVent and ventilated at 450 breaths/min with a tidal volume of 8 mL/kg and a PEEP of 2 cmH_2_O. Following standardisation of the lung volume history via three slow deep inflations to a respiratory system pressure of 20 cmH_2_O, respiratory mechanics at functional residual capacity were determined using the low frequency FOT and fitting of the constant phase model to Z_rs_. Measurements were taken once per min for 5 min. Then a 10 s challenge of saline aerosol, and increasing doses (0.1, 0.3, 1, 3, 10 and 30 mg/mL) of methacholine (Acetyl β-methacholine chloride, Sigma–Aldrich, MO, USA) were delivered by an ultrasonic nebulizer (DeVilbiss UltraNeb, Somerset, PA, USA). Respiratory system impedance was then measured once per min for 5 min after each challenge with data used to create dose response curves.

### Bronchoalveolar lavage fluid for assessment of inflammatory cells

Bronchoalveolar lavage fluid (BALF) was collected by slowly washing the lungs with saline (250 μL for offspring and 500 μL for dams) via the endotracheal tube three times. BALF was centrifuged at 400 x g (Centrifuge 5415D, Eppendorf) and BALF supernatant stored at − 20 °C. The pellet was resuspended and mixed in PBS (100 μl), and the total concentration of cells in BALF determined using a haemocytometer (Neubauer Chamber). BALF cells (5 × 10^5^) were spun onto glass slides using a cytocentrifuge and differential counts of inflammatory cells performed after staining cells with the DIFF-Quik Stain Set 64,851 (Lab Aids, Narrabeen, NSW, Australia) as per the manufacturer’s instructions. At least 300 cells were counted for each sample from ≥3 independent fields of view (× 100) in a blinded fashion.

### Measurement of cytokine and adipokine protein levels in BALF

TNFα, IL-5 and IL-33 levels were measured in BALF of offspring and dams using a modified ELISA/time-resolved fluorescence method, as previously described [30]. The limits of detection of the TNFα, IL-5 and IL-33 assays were 7, 8 and 9 pg/mL, respectively. Adiponectin and leptin levels were measured in the BALF of offspring and dams using ELISA kits (EMD Millipore Corporation, Billerica, MA) as described by the manufacturer. The limits of detection of the adiponectin and leptin assays were 0.2 and 0.05 ng/mL, respectively.

### Lung structure

Following euthanasia, a subset of lungs from offspring were inflation fixed with 10% buffered-formalin (Sigma-Aldrich, MO, United States of America) in PBS via the endotracheal tube, removed *en bloc* and left overnight before being transferred to saline (AstraZeneca, NSW, Australia) (at 4 °C) [31]. Fixed lungs were embedded in paraffin, and the left lobe was sectioned for assessment of mean linear intercept (chord) length (L_m_). A lung section was selected at random from each individual. It was then masked and 20 fields of view were randomly generated using stereological software (newCAST, Visiopharm, Hørsholm, Denmark). As per established techniques, all straight-line segments that spanned the air space between two sequential intersections of the alveolar surface on three random test lines were measured in each field of view [32], resulting in at least 250 Lm measurements per mouse.

### White adipose tissue weight

Gonadal white adipose tissue (WAT) was obtained from dams and weighed using an electronic balance (OHAUS, analytical standard, level of sensitivity > 0.1 mg).

### Statistical analyses

Data comparing outcomes from dams fed a high or low fat diet were compared using an unpaired two-way student’s *t* test for normally distributed data, or Mann-Whitney test with non-normally distributed data (determined using the D’Agostino-Pearson omnibus normality test), using Prism 5 for Mac OS X. Area under the curve was calculated for GTT using GraphPad Prism (v5) using 0 as the baseline. Data comparing outcomes of pregnancies 1 or 2, for dams or offspring born to dams fed a high or low fat diet, were compared using two-way ANOVA with data transformed to satisfy the assumptions of normality and homoscedasticity where required, using SigmaPlot for Windows v13.0. Data from male and female pups were combined due to the low number of surviving 2 week-old offspring, which were born to dams fed a high fat diet. A Pearson’s correlation test was used to determine the significance and strength of linear relationships between TGV and body length. Differences were considered significant with a *p-*value < 0.05. For some analyses, a two-tailed Fisher’s Exact test with a confidence interval of 95% was used. Data are shown as mean ± standard error of the mean (SEM).

## Results

### Dams fed a high fat diet gained weight and displayed signs of metabolic dysfunction

**Fig. 2:**
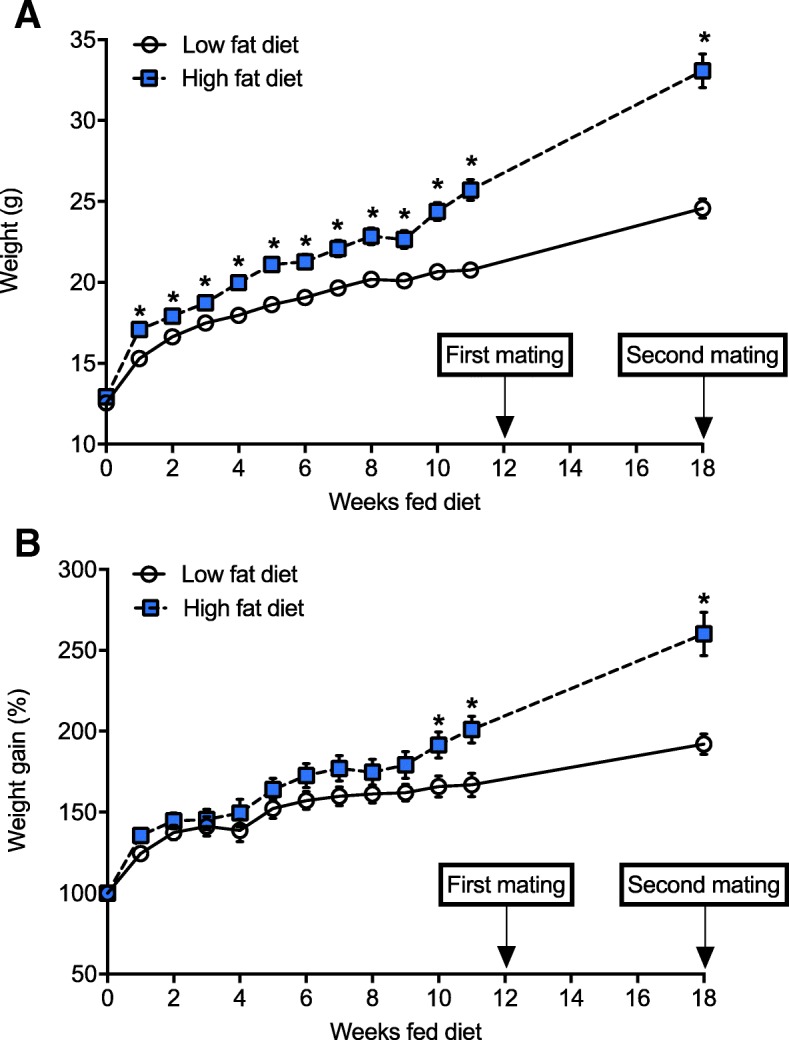
Female C57Bl/6 J gained more weight when fed a high fat diet*.* From 4 weeks of age, female mice were fed either a low fat diet (LFD, *n* = 14) or high fat diet (HFD, n = 14). Female mice were mated with male mice, first after dams were fed either diet for 12 weeks, and secondly 1 week after the first litter of 2 week-old offspring were tested for lung function. Dams were weighed on a weekly basis until mice were mated for the first time, with a further weighing immediately prior to the second mating. Data are shown as mean ± SEM for n = 14 mice/treatment. In (**a**), body weights and in (**b**) percentage weight gain are shown (*p < 0.05)

**Fig. 3:**
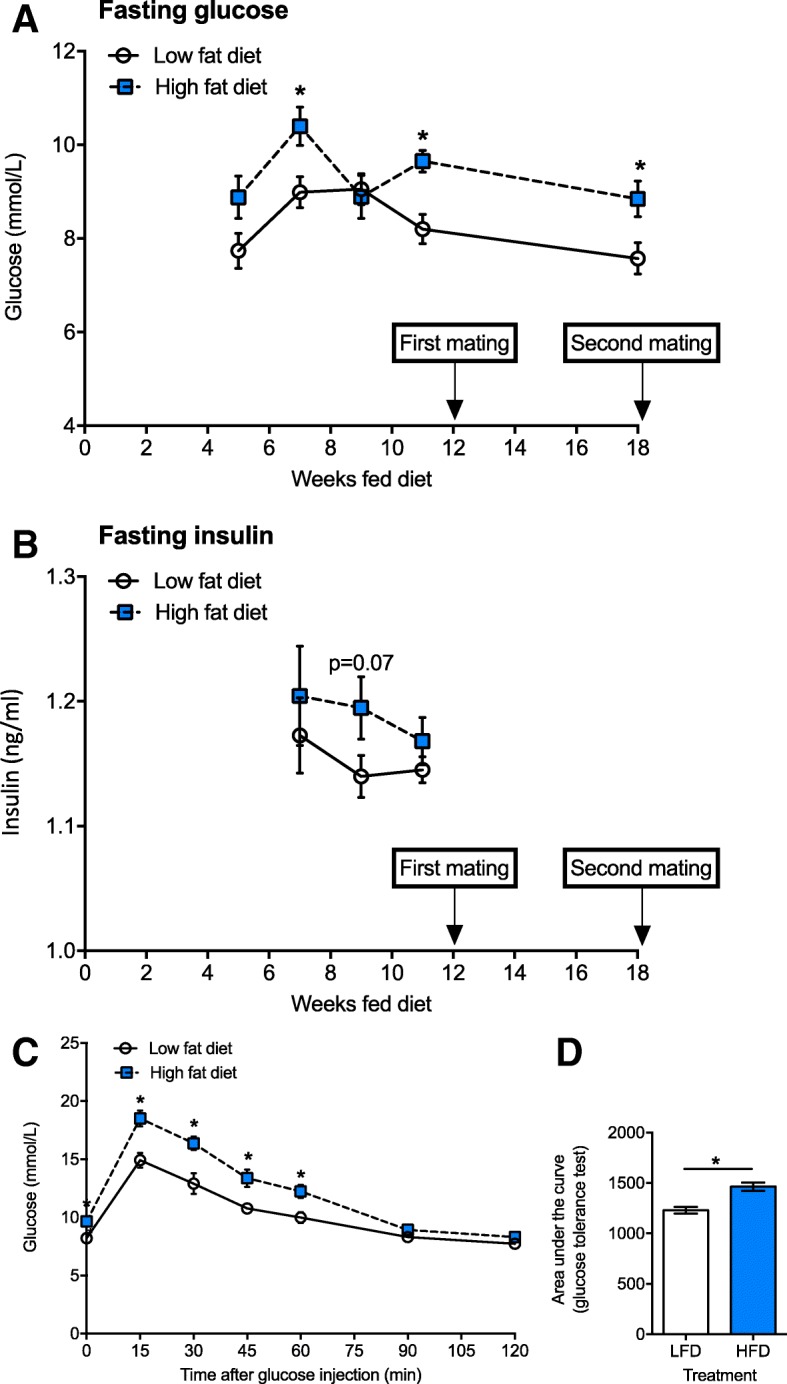
Female C57Bl/6 J mice fed a high fat diet developed metabolic dysfunction*.* From 4 weeks of age, female mice were fed either a low fat diet (LFD, *n* = 14) or high fat diet (HFD, n = 14). Female mice were mated with male mice, firstly after dams were fed either diet for 12 weeks, and secondly 1 week after the first litter of 2 week-old offspring were tested for lung function. In (**a**), fasting glucose at 5, 7, 9, 11 and 18 weeks after consuming either diet. In (**b**), fasting insulin at 7, 9, and 11 weeks after consuming either diet. In (**c**), results of glucose tolerance tests are shown for dams tested 11 weeks after consuming either diet, with glucose levels at various times post-injection of mice with glucose (1 g glucose/kg body weight), and (**d**), the area under the curve for these tests. Data are shown as mean ± SEM for n = 14 mice/treatment (*p < 0.05)

Female C57Bl/6 J mice had increased body weight (Fig. 2a) after 1 week, and weight gain (Fig. 2b) after 10 weeks of consuming the high fat diet. Female mice fed the high fat diet continued to gain weight throughout the experiment to a greater extent than mice fed the low fat diet (Fig. 2). Significantly increased fasting glucose was observed at 7, 11 and 18 weeks after mice began eating the high fat diet, compared with dams fed a low fat diet (Fig. 3a). There was some evidence (Student’s *t* test; *p* = 0.07) for increased fasting insulin in female mice fed the high fat diet for 9 weeks (compared with those fed the low fat diet); however, the difference between treatments was small (< 0.1 ng/ml Fig. 3b). In the week prior to first mating (Fig. 1), results of a GTT indicated that dams fed the high fat diet had developed signs of glucose intolerance, with increased glucose levels observed throughout the GTT, with increased area under the curve of the GTT in mice fed a high fat diet compared with control mice (Fig. 3c, d).

### Fewer litters born to dams fed a high fat diet survived until 2 weeks of age

For either pregnancy, there was no difference in the number of mice with confirmed pregnancies, the time for pregnancies to be confirmed or weight gain during the first 10 days of pregnancy, for dams fed a high or low fat diet (Table 2). The number of litters that survived until 2 weeks of age was significantly reduced for dams fed the high fat diet for both pregnancies, with no differences in the number of pups or sex ratio of pups born to each litter (Table 3). However, when litter size data were combined for the first and second pregnancies, there were fewer male (1.0 ± 0.4, mean ± SEM) than female (2.8 ± 0.2) offspring born to dams fed a high fat diet (Student’s *t* test; *p* = 0.01), which was not observed in the low fat diet treatment (data not shown).

**Table 2:** Eating a high fat diet did not affect the number of pregnancies or time to confirmed pregnancy or initial pregnancy weight gain of female C57Bl/6 J mice

Outcome	LFD	HFD
First pregnancy		
Confirmed pregnancies (n)	14/14	14/14
Time to confirmed pregnancy (days)	10.5 ± 0.8	10.9 ± 0.8
Weight gain - first 10 days of pregnancy (g)	4.8 ± 0.5	5.2 ± 0.6
(%)	123 ± 3	122 ± 3
Second pregnancy		
Confirmed pregnancies (n)	8/14	8/14
Time to confirmed pregnancy (days)	9.8 ± 0.4	8.4 ± 0.6
Weight gain - first 10 days of pregnancy (g)	5.0 ± 0.6	5.3 ± 0.5
(%)	124 ± 3	122 ± 3

From 4 weeks of age, C57Bl/6 J female mice (*n* = 28) were separated into 2 treatment groups and fed either a low fat diet (LFD, *n* = 14) or high fat diet (HFD, *n* = 14). Female mice were first mated with C57Bl/6 J male mice after dams had been fed either diet for 12 weeks. Three weeks later, offspring were born. Dams were re-mated 1 week after their first litters were removed at 2 weeks of age. Data are shown as mean ± SEM

**Table 3:** Fewer litters born to dams fed a high fat diet survived until 2 weeks of age

Outcome	LFD	HFD
First pregnancy		
Litter survival (n)	9/14	2/14*
Pups per litter (n)	2.9 ± 0.6	4.0 ± 1.0
Male pups per litter (n)	1.4 ± 0.4	1.0 ± 1.0
Female pups per litter (n)	1.3 ± 0.4	3.0 ± 0.0
Second pregnancy		
Litter survival (n)	7/8	2/8*
Pups per litter (n)	4.7 ± 0.6	3.5 ± 0.5
Male pups per litter (n)	1.9 ± 0.4	1.0 ± 0.0
Female pups per litter (n)	2.9 ± 0.5	2.5 ± 0.5

*p < 0.05, compared with dams fed a LFD (using Fisher’s; Exact Test)

From 4 weeks of age, C57Bl/6 J female mice (*n* = 28) were separated into 2 treatment groups and fed either a low fat diet (LFD, *n* = 14) or high fat diet (HFD, n = 14). Female mice were first mated with C57Bl/6 J male mice after dams had been fed either diet for 12 weeks. Three weeks later, offspring were born. Dams were re-mated 1 week after their first litters were removed at 2 weeks of age. The numbers of offspring (male or female) per litter are reported for pups that survived until 2 weeks of age. Data are shown as mean ± SEM

### Offspring born to dams fed a high fat diet were bigger

Due to limited survival of offspring, particularly born to dams fed a high fat diet (only 2 litters for each mating, *n* = 14 dams), we were not able to select to report data from a single male and female offspring of each litter as suggested by some researchers to avoid the “litter effect” [33]. Surviving two-week-old offspring born to dams fed a high fat diet weighed more, were longer (snout to vent length) and had increased body mass index (BMI, kg/m^2^) than control offspring (*p* < 0.001, Table 4). In addition, offspring born from second pregnancies weighed more and were longer than offspring of the first pregnancies (*p* < 0.035).

**Table 4:** Offspring fed a high fat diet had increased body weights and lengths

Outcome	LFD	HFD
First pregnancy	*n* = 18	*n* = 8
Body weight (g)	5.8 ± 0.2	7.4 ± 0.8*
Body length (snout to vent) (mm)	48.0 ± 0.8	53.5 ± 1.4*
Body mass index (kg/m^2^)	2.48 ± 0.06	2.53 ± 0.17*
Second pregnancy	*n* = 33	*n* = 7
Body weight (g)	5.9 ± 0.1	8.9 ± 0.3*†
Body length (snout to vent) (mm)	49.1 ± 0.6	56.7 ± 1.0*†
Body mass index (kg/m^2^)	2.47 ± 0.04	2.77 ± 0.09*§

There was a significant effect of both diet (p < 0.001*) and pregnancy (*p* < 0.035†) on offspring body weight and length (2-way ANOVA) with no significant interactions (*p* < 0.299). There was a significant effect of diet only (*p* < 0.001§) but not pregnancy (*p* = 0.169) on offspring BMI (2-way ANOVA) with no significant interaction (*p* = 0.131)

From 4 weeks of age, C57Bl/6 J female mice (*n* = 28) were separated into 2 treatment groups and fed either a low fat diet (LFD, *n* = 14) or high fat diet (HFD, n = 14). Female mice were mated with male C57Bl/6 J mice after dams were fed either diet for 12 weeks. Three weeks later, offspring were born. Dams were re-mated 1 week after their first litters were removed at 2 weeks of age. All measures were obtained from offspring at 2 weeks of age. Data are shown as mean ± SEM

### Lung function of offspring

**Fig. 4:**
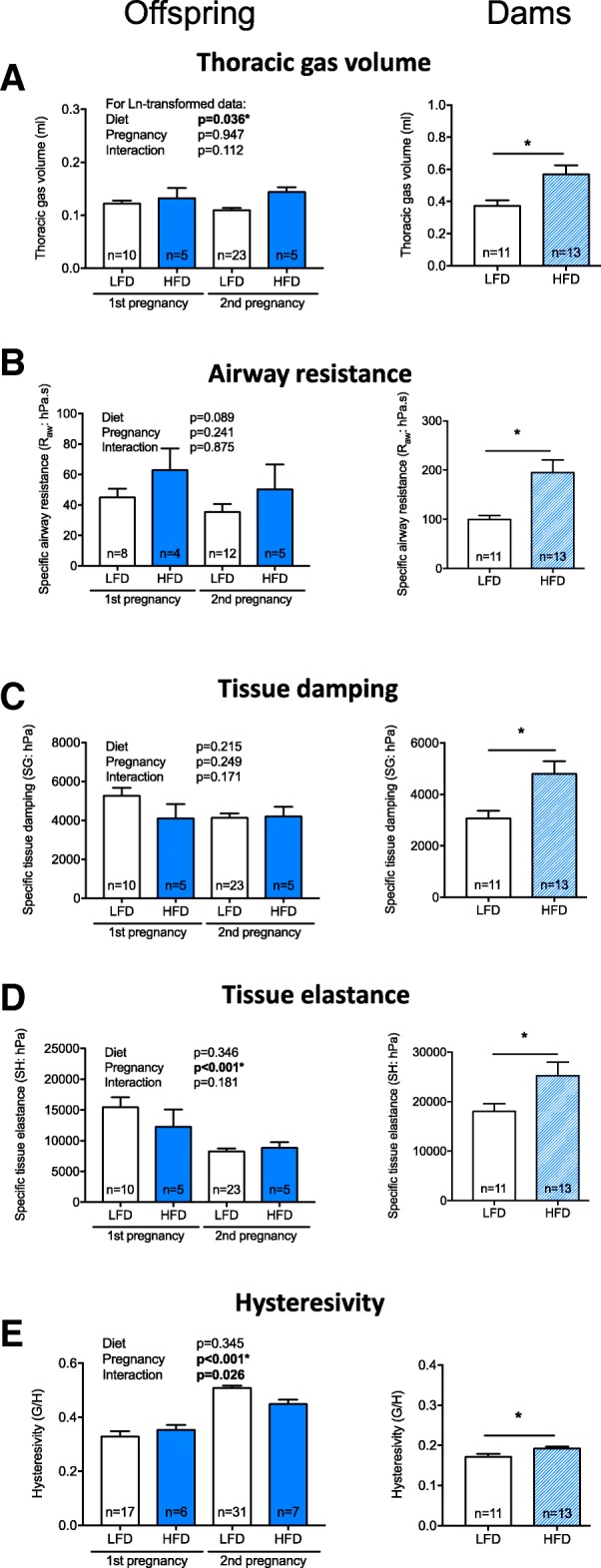
The lung volume of offspring and dams was increased by maternal consumption of a high fat diet. From 4 weeks of age, female mice were fed either a low fat diet (LFD, *n* = 14) or high fat diet (HFD, n = 14). Female mice were mated with male mice, first after dams were fed either diet for 12 weeks, and secondly 1 week after the first litter of offspring was tested for lung function. Offspring of both pregnancies had their lung function measured at 2 weeks of age. The dams had their lung function measured after eating the LFD or HFD for 29 weeks. In (**a**), thoracic lung volume; (**b**), specific airway resistance (SR_aw_); (**c**), specific tissue damping (SG); (**d**), specific tissue elastance (SH); and (**e**), hysteresivity (G/H) measured for offspring born to the first and second matings, and dams (respectively). Data are shown as mean + SEM, with number of mice (n)/treatment shown (*p < 0.05)

Offspring born to dams that ate a high fat diet had larger lung volumes (TGV) compared with offspring born to dams that ate a low fat diet (*p* = 0.036, Fig. 4a). As a result we deemed it more appropriate to compare parameters of lung function corrected for lung volume. There was no significant effect of mother’s diet on specific airway resistance (SR_aw_; *p* = 0.089, Fig. 4b), specific tissue damping (SG; *p* = 0.215, Fig. 4c), specific tissue elastance (SH; *p* = 0.346, Fig. 4d) or hysteresivity (*p* = 0.345, Fig. 4e). Similarly, there was no effect of pregnancy number (1st or 2nd) on TGV (Fig. 4a), specific airway resistance (SR_aw_, Fig. 4b) or tissue damping (SG, Fig. 4c) (*p* > 0.241 in all cases) (Fig. 4). Pups born from the second pregnancies had significantly lower specific tissue elastance (SH) (*p* < 0.001, Fig. 4d) and higher hysteresivity (*p* < 0.001, Fig. 4e) compared with pups born from the first pregnancy. There was a significant interaction between diet and pregnancy for hysteresivity, such that pups born to mice fed the low fat diet and born from the second pregnancies had increased hysteresivity compared with all other groups (*p* = 0.026, Fig. 4e). Interestingly, when we examined the relationships between TGV and body length, while we did not observe significant linear relationships between TGV and body length in offspring born to mothers fed a high fat diet (Pregnancy 1: *r* = 0.46, *p* = 0.43; Pregnancy 2: *r* = 0.51, *p* = 0.38; Pregnancy 1&2: *r* = 0.50, *p* = 0.14, Pearson’s) or low fat diet (Pregnancy 1: *r* = 0.61, *p* = 0.06; Pregnancy 2: *r* = 0.22, *p* = 0.31; Pregnancy 1&2: *r* = 0.12, *p* = 0.28), when results from all offspring were combined there was a moderately strong and significant linear relationship between TGV and body length (*r* = 0.47, *p* = 0.001). These findings suggest that TGV may be influenced by body length.

### Lung function of dams

**Fig. 5:**
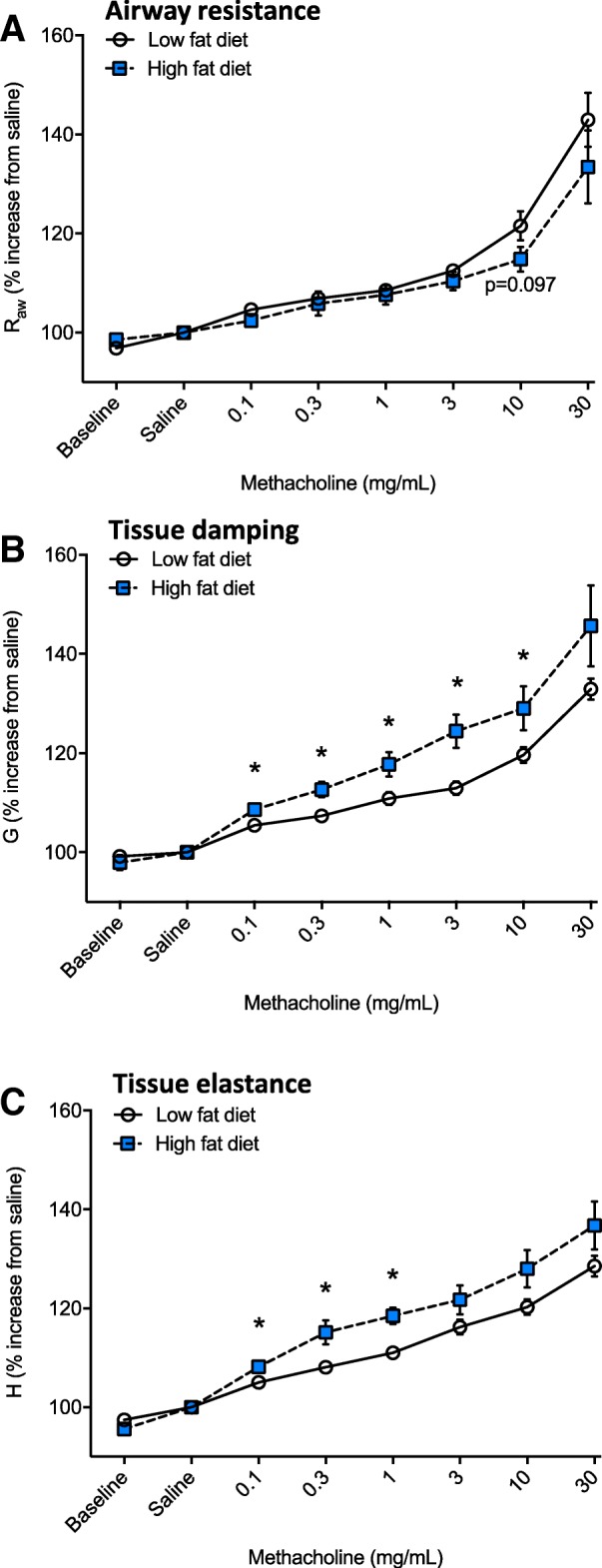
Dams fed a high fat diet for 29 weeks were more responsive to methacholine*.* From 4 weeks of age, female mice were fed either a low fat diet (LFD, n = 14) or high fat diet (HFD, *n* = 13). Female mice were mated with male mice, first after dams were fed either diet for 12 weeks, and secondly 1 week after the first litter of 2 week-old offspring were tested for lung function. After eating the LFD or HFD for 29 weeks, the dams had their lung function measured following an initial challenge with saline and then increasing doses of methacholine. In (**a**), airway resistance; in (**b**), tissue damping; and, in (**c**), tissue elastance are shown. Data are shown as mean ± SEM (*p < 0.05)

By 33 weeks of age, dams fed a high fat diet for 29 weeks weighed significantly more (*p* = 3.5 × 10^− 7^), had longer body lengths (*p* = 2.4 × 10^− 7^), increased BMI (*p* = 1.9 × 10^− 4^) and substantially heavier deposits of gonadal WAT (*p* = 1.1 × 10^− 9^) than dams fed the low fat diet (Table 5). When their lung function was assessed, dams fed a high fat diet exhibited increased lung volume (*p* = 0.011, Fig. 4a). Similar to findings from offspring mice, although not quite significant, there was some evidence for a linear relationship between TGV and body length when results for dams fed either diet were combined (*r* = 0.40, *p* = 0.05). At functional residual capacity dams fed a high fat diet also had significantly higher specific airway resistance (SR_aw_, *p* = 0.004, Fig. 4b), tissue damping (SG, *p* = 0.01, Fig. 4c), tissue elastance (SH, *p* = 0.04, Fig. 4d) and hysteresivity (*p* = 0.03, Fig. 4e) compared with dams fed a low fat diet. There was no significant effect of eating a high fat diet on responsiveness to methacholine with respect to airway resistance (Fig. 5a); however, increased tissue damping (*p* ≤ 0.017, Fig. 5b) and tissue elastance (*p* ≤ 0.01, Fig. 5c) responses were observed in dams fed a high fat diet.

**Table 5:** Dams exhibited signs of obesity when fed a high fat diet for 29 weeks

Outcome	LFD	HFD
Body weight (g)	26.1 ± 0.5	38.5 ± 1.8*
Body length (snout to vent) (mm)	88.9 ± 0.8	96.4 ± 0.7*
Body mass index (kg/m^2^)	3.30 ± 0.08	4.14 ± 0.18*
Gonadal white adipose tissue (g)	0.52 ± 0.10	3.84 ± 0.35*

*p < 0.05, compared with dams fed a LFD (student’s *t* test)

From 4 weeks of age, C57Bl/6 J female mice (n = 28) were separated into 2 treatment groups and fed either a low fat diet (LFD, *n* = 14) or high fat diet (HFD, n = 14) for 29 weeks. Female mice were mated with male C57Bl/6 J mice after dams were fed either diet for 12 weeks. Three weeks later, offspring were born. Dams were re-mated 1 week after their first litters were removed at 2 weeks of age. Data are shown as mean ± SEM for *n* = 13–14 dams/treatment

### Increased signs of inflammation were observed in dams (but not offspring) fed a high fat diet

**Fig. 6:**
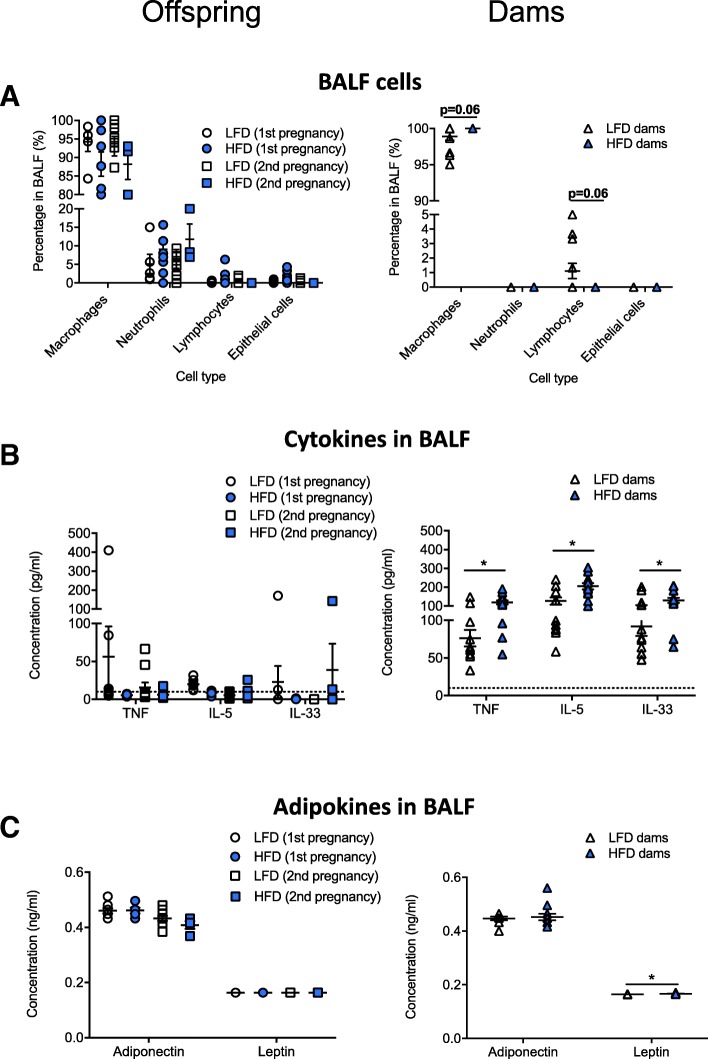
Increased TNF, IL-5 and IL-33 are observed in the lungs of dams fed a high fat diet, but not their offspring*.* From 4 weeks of age, female mice were fed either a low fat diet (LFD, n = 14) or high fat diet (HFD, n = 13). Female mice were mated with male mice, first after mice were fed either diet for 12 weeks, and secondly 1 week after the first litter of 2 week-old offspring were tested for lung function. The proportions of: (**a**) major cell types; (**b**) levels of TNF, IL-5, and IL-33; and, (**c**) adiponectin and leptin were measured in the BALF of offspring (2 week-old, *n* ≥ 5) and dams fed a LFD or HFD for 29 weeks. Data are shown as mean ± SEM (*p < 0.05), with some data points overlapping (particularly for part (A) dams)

There was no effect of diet on the type of inflammatory cells in the BALF of offspring with > 80% of cells identified as macrophages, and neutrophils the next most prevalent cell (Fig. 6a). In dams fed a high fat diet, there were trends (*p* = 0.06, two way student’s *t* test) for increased proportions of macrophages, and reduced proportions of lymphocytes, with few neutrophils detected in BALF (Fig. 6a). Significantly increased levels of TNFα, IL-5, IL-33 and leptin were detected in BALF of dams fed a high fat diet compared with dams fed a low fat diet (*p* < 0.05, Fig. 6b, c). Levels of TNFα, IL-5, IL-33 in the BALF of offspring were frequently at or below the levels of detection of the ELISAs (Fig. 6b), with no effect of maternal diet on adiponectin or leptin levels in offspring (Fig. 6c). Adiponectin levels were reduced in the BALF of offspring born from the second pregnancies compared with the first (Fig. 6c; two-way ANOVA, *p* = 0.0013).

### Mean linear intercept

Morphological analysis of lungs from offspring revealed that there was a trend for reduced mean linear intercept length (the mean free distance of the air spaces in the lungs) in offspring born to dams fed a high fat diet (27.3 ± 2.0, mean ± SEM, *n* = 6), compared with control offspring (33.5 ± 2.2, mean ± SEM, *n* = 4; *p* = 0.08, two-way student’s *t* test).

### Reduced IL-33 and leptin levels were observed in the BALF of dams fed a high fat diet that were pregnant on two occasions

**Fig. 7:**
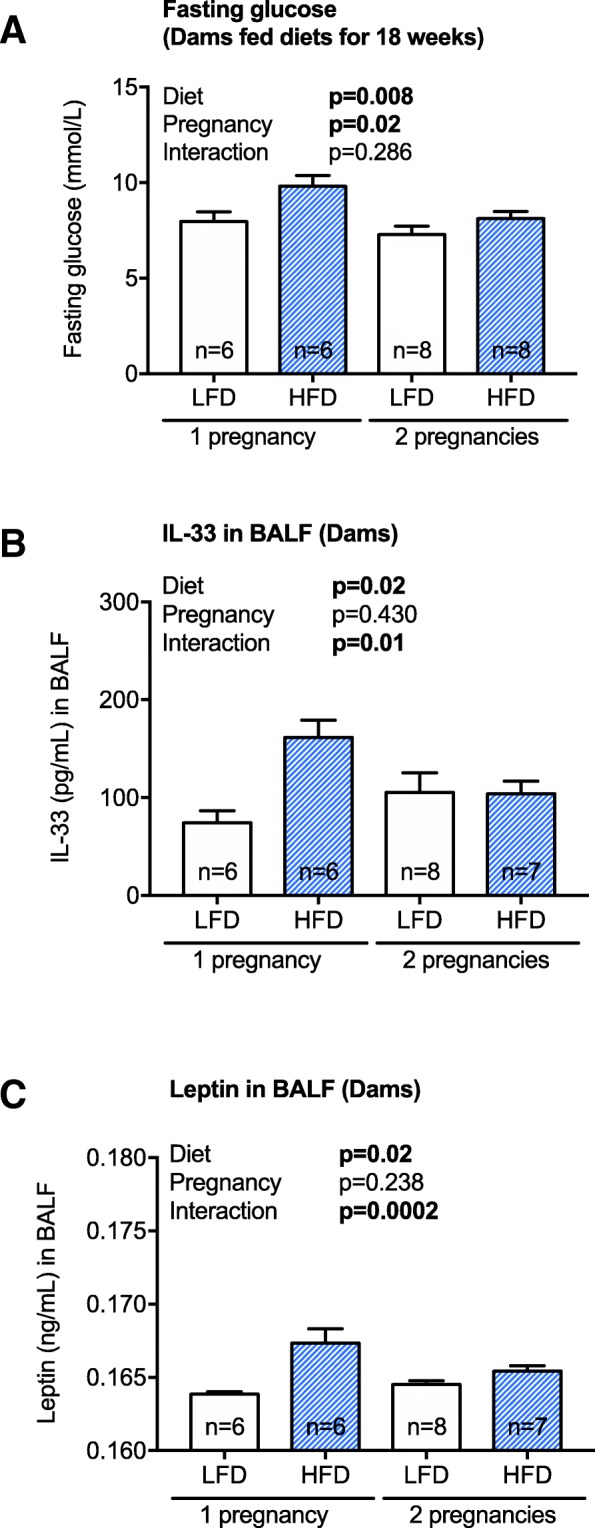
Dams fed a high fat diet had reduced fasting glucose levels prior to a second pregnancy, and BALF levels of IL-33 and leptin at the experimental endpoint. From 4 weeks of age, female mice were fed either a low fat diet (LFD, *n* = 14) or high fat diet (HFD, *n* = 13). Female mice were mated with male mice twice, after mice were fed either diet for 12 and then 18 weeks, with (**a**) fasting glucose measured immediately prior to second mating. BALF levels of (**b**) IL-33 and (**c**) leptin, from dams fed a LFD or HFD for 29 weeks, were measured. Data are shown as mean ± SEM (*p < 0.05)

Considering that there were effects of pregnancy number on some outcomes detailed above, and new reports describing that an initial pregnancy may prime immune responses experienced in subsequent pregnancies [34], we re-examined outcomes, separating results for dams that had 1 or 2 pregnancies. When fasting glucose levels were measured immediately prior to the second mating (Fig. 3a, in dams fed the high or low fat diets for 18 weeks), those dams able to become pregnant a second time had significantly reduced levels at this 18-week time-point (Fig. 7a), suggestive of an adaptation to the effects of eating a high fat diet. There were no significant differences in the lung function of dams that were pregnant on 1 or 2 occasions (data not shown, *p* > 0.05). Similarly, there was no significant effect of pregnancy on the types of cells or concentration of cytokines or adipokines measured in the BALF of dams (data not shown, *p* > 0.05). However, there were significant interactions between diet and pregnancy, such that those mice that ate a high fat diet, and were pregnant twice, had reduced BALF levels of IL-33 (Fig. 7b; two-way ANOVA, *p* = 0.01) and leptin (Fig. 7c; two-way ANOVA, *p* = 0.0002), compared to those fed a high fat diet and were pregnant only once.

## Discussion

In this study, we examined the effects of maternal consumption of a high fat diet during pre-conception, pregnancy and lactation on the lung function of 2 week-old offspring. The high fat diet significantly increased body weight, weight gain and WAT deposits in female mice (dams-to-be), and induced metabolic dysfunction (elevated fasting glucose and glucose intolerance) prior to breeding. Reduced litter survival was observed for pups born to dams fed the high fat diet. Those pups that did survive until 2 weeks of age were longer, weighed more, and had increased lung volumes compared with offspring born to dams fed the low fat diet. However, the high fat diet did not compromise the lung function of offspring, with no significant effect on specific airway resistance, tissue damping or tissue elastance. Eating the high fat diet also increased the lung volume of dams, as well as specific airway resistance, tissue damping and elastance (Fig. 4) and responsiveness to methacholine (tissue damping and elastance, Fig. 5). No overt signs of inflammation were observed in the BALF of offspring of dams fed a high fat diet, while increased TNFα, IL-5, IL-33, and leptin levels were observed in BALF of their mothers. These findings suggest that maternal consumption of a high fat diet during the preconception, pregnancy and lactation periods increases somatic growth, which is likely linked with increased lung volume of both the offspring and dams, and that long-term consumption of a high fat diet modifies lung function and promotes respiratory inflammation in adulthood.

While significant increases in lung volume and body size (weight and length) were observed in offspring born to dams fed a high fat diet, we also observed a non-significant increase in airway resistance (*p* = 0.089). This failure to achieve significance may have been caused by the low sample size (an effect of the maternal high fat diet on offspring survival), with the 30% difference likely to be biologically significant (HFD mean SRaw is 55.9 hPa.s; LFD mean SRaw is 39.3 hPa.s). We also observed some non-significant reductions in the mean linear intercept length of offspring born to dams fed the high fat diet (p = 0.08), perhaps indicative of more fully developed lungs. In the mouse, 50% of alveolar septa form between days 4 and 14 of life with a further 40% between days 14 and 36 [35], suggesting that our measurements of lung function at 2 weeks of life, were done during a period of rapid alveolarization. Interestingly, recent studies suggest ‘airway dysanapsis’ may exist in obese children, which is defined as the ‘incongruence between lung and airway growth’ [36, 37]. In obese children, airway dysanapsis is demonstrated by increased lung volume, and reduced airflow [36, 37]. Our observations of increased lung volume and a trend towards increased airway resistance are suggestive of a similarity to those of airway dysanapsis in obese children, although it is important to note that the stage of development of lungs in young mice and children may not be equivalent, as most alveolarization occurs post-birth in the lungs of mice (unlike humans) (reviewed by [38]). Together, these studies indicate that the increased nutrition supplied during exposure to high fat diet in utero and lactation increases lung (and body) size, with a reduction in mean linear intercept suggestive of ‘more fully developed’ lungs but has detrimental effects on other aspects of lung development. A future goal could be to determine if there is a window of high fat diet exposure (e.g. pre-conception, gestation, lactation), which is responsible for increasing lung volume in offspring.

Clinically, the ‘obese asthma’ phenotype in childhood is characterized by increased severity, reduced disease control and impaired quality of life (compared with age-matched, non-obese individuals) [39]. Obese asthma in children may not be associated with airway or systemic inflammation [6]. Similarly, we did not observe any signs of inflammation (or mucus) in the lungs through histopathological assessment (data not shown) or measurement of cytokines and adipokines in BALF in offspring born to dams fed a high fat diet. Indeed, levels of TNFα, IL-5 and IL-33 were below the level of detection of the assay in many samples. In contrast, obese asthma in adults is associated with increased airway and systemic inflammation [6], consistent with our observations of increased TNFα, IL-5, IL-33 and leptin concentrations in the BALF of dams fed the high fat diet. It might be that further exposure to a high fat diet, and/or other stimuli (e.g. viral infection/allergen), with immune maturation, are required for offspring to produce significant cytokine levels in the lung. In similar studies, exposure to high fat diet during lactation (only) induced an early-onset obesity in offspring (C57BL/N mice) at 3 weeks of age, with increased expression of mRNAs of IL-5, IL-13, IL-17A and TNFα in the lung [40]. Interestingly, the effect of exposure to the high fat diet during lactation was transient, with no difference in these cytokine mRNAs observed in the lungs of offspring when they reached 10 weeks of age, and limited effects on peribronchial elastic fibre content, bronchial smooth muscle mass and connective tissue deposits in the lungs [40]. Even so, increased airway hyperresponsiveness to methacholine was observed in offspring exposed to the high fat diet during lactation [40], suggesting that ‘short-term’ exposures (such as that experienced during lactation) can have long-lasting effects on lung function.

Other studies suggest that pro-inflammatory cytokines/adipokines can affect surfactant production in the lungs. Indeed, increased expression of TNFα and leptin have been linked with reduced surfactant production [6]. In utero exposure to high fat diet or excessive nutrition can reduce surfactant expression in the lungs of foetal mice (embryonic day 18 [13]), foetal sheep (141 days gestation) [15]) and neonatal rats (2 day-old [14]). The effects of high fat diet in inducing airway dysfunction and inflammation may not be limited to exposures during pregnancy, with post-weaning intake of a high fat diet increasing airway hyperresponsiveness to methacholine, and promoting neutrophils and higher IL-6 levels in the BALF in 10 week-old BALB/cByJ mice previously fed a high fat diet from 3 weeks of age [41]. Ongoing early life and post-weaning exposure to high fat diet can also promote fibrosis in the lungs, with increased transforming growth factor-ß and α-smooth muscle actin protein levels observed in the lungs of 12 week-old offspring born to Sprague-Dawley dams fed a high fat diet for 9 weeks before mating [42]. Reduced concentrations of nitric oxide metabolites and enhanced arginase levels were also observed in the lungs of adult offspring of C57Bl/6 J dams fed a high fat diet for 6 weeks prior to mating [43]. These observations may explain the increased tissue elastance (lung stiffness) responses observed in dams fed a high fat diet, following methacholine challenge. Together, these findings suggest that multiple interacting pathways are induced by maternal exposure to high fat diet to cause lung dysfunction and potentially increase susceptibility for obese asthma in offspring.

There are two types of adult asthma induced by obesity: 1) early onset asthma complicated by obesity (Th2 (Th2)-skewed) [39]; and, 2) late-onset with a low Th2 phenotype. This late-onset phenotype is linked with non-atopic asthma in adults [44], and has a distinct clinical phenotype, characterized by neutrophilic airway and systemic inflammation [45], obstructed and restricted lung function, increased severity and hospitalization rates, reduced disease control and quality of life and impaired responsiveness to corticosteroids [39, 44]. Our observations of increased Th1 (TNFα) and Th2 (IL-5, IL-33) cytokine concentrations in the BALF of dams fed a high fat diet, without any neutrophilia or eosinophilia suggest that these mice are ‘poised’ to react to further allergenic or viral exposure. However, our findings of increased lung volume in dams fed a high fat diet are not aligned with ongoing hypotheses of the effects of obesity on lung function in humans, in which it is thought that excessive accumulation of fat in the thoracic and abdominal compartments compresses the lungs to reduce lung volume and increase risk for peripheral airway and parenchyma collapse [6, 39]. These differences in the effects of obesity on lung volume measurements in mice compared with human (as adults), could be explained by differences in the orientation of mice vs humans (supine vs sitting) when lung function is being assessed. Our means of testing lung function, with mice in the supine position under general anaesthetic may allow for more extensive lung inflation than achievable in humans.

Another interesting finding of our study was an independent effect of second pregnancy on offspring weight and lung function outcomes. Offspring from pregnancy two weighed more than offspring of pregnancy one (regardless of the diet of the mother). It is possible that the increasing age of female mice increased the risk for pregnancy complications that could contribute towards increased body weight of offspring. Older women are at increased risk for gestational diabetes, independently of pre-gestation BMI and obesity [46]. We did not measure any metabolic outcomes during either pregnancy due to concerns that even small interventions in the dam could affect litter survival especially those fed the high fat diet. However, reduced fasting glucose levels were observed in dams fed a high fat diet that were able to become pregnant a second time (immediately prior to mating), suggesting that some adaption to the effects of high fat diet may be necessary to become pregnant a second time (although most of these pregnancies were not successful). It may be that glucose metabolism was further adversely affected in the second pregnancy. Furthermore, maternal age, and multiparity may interact to produce different maternal environments that modify the lung development of offspring. Conversely, other studies suggest that immune training experienced in the first pregnancy may support better placentation events in second pregnancies through the activity of specific subset of natural killer cells and increased expression of molecules that support vascularization (e.g. VEGFα) [34]. Similarly, we also observed reduced levels of the innate Th2 cell-promoting cytokine IL-33, and the adipokine, leptin in the lungs of dams fed a high fat diet that were pregnant twice. Interestingly, increased expression of these mediators has been shown to promote asthma exacerbation and airway hyper-reactivity in animal models [47]. Furthermore, reduced tissue elastance (and adiponectin levels in BALF) was observed in the offspring of the second pregnancy of dams fed either diet; suggesting that there may be protective effects of a second pregnancy that extend to the offspring in some developmental niches (i.e. the lungs).

Others have also recorded adverse effects of maternal high fat diet consumption on foetal and neonate survival. A 2-fold reduction in foetal survival (at embryonic day 18) was observed in pregnant C57Bl/6 J dams fed a high fat diet for 3–5 months prior to mating [13]. Increased perinatal death rates were observed in Sprague-Dawley dams fed a high fat diet for 4 weeks prior to mating [14]. Obesity and excessive weight gain also increases risk for early miscarriage, complications and stillbirth in human pregnancies [48, 49]. Han et al. (2017) observed in ICR mice, that maternal consumption of a high fat diet impaired the expression of the Stella protein in oocytes, resulting in pronuclear epigenetic asymmetry in zygotes and DNA damage, increasing rates of foetal and neonatal death, and promoting the development of aberrant metabolic phenotypes in surviving offspring [50]. It may be that high fat diet consumption also has epigenetic effects in genes essential for optimal lung development.

Our initial reasoning for feeding dams the high fat diet for 12 weeks was to demonstrate metabolic dysfunction (e.g. increased glucose intolerance, Fig. 3) in addition to increased body weight prior to mating mice. At the time of our experiments (early 2015), there were only a few reports published describing some moderate adverse effects of high fat diet on offspring survival. For example, Krasnow et al (2011) reported higher mortality (23%, compared to 3% for low fat diet) for pups born to second pregnancies for C57Bl/6 J dams fed a high fat diet for 12 weeks (prior to mating) [51]. In a model very similar to our own, King et al (2013) reported that fewer pups were born to dams fed a high fat diet for 12 weeks prior to and during mating, as well as lactation, compared to dams fed a low fat diet (high fat diet = 5.0 pups/litter (mean), control diet = 6.5 pups/litter (mean) [52]. However, the effects of our maternal high fat diet model on offspring survival were more severe (Table 3). The reasons for this difference are unclear, although they could have been mediated through differences in animal housing or diet (our high fat diet was 46%, versus 58% digestible lipid for [52]). Alternatively researcher-based issues may account for the observed differences between these studies. Indeed, handling of mice by male researchers may be more stressful for mice [53], and the person mainly handling animals in our study was male. This may have contributed towards higher than expected rates of litter death (potentially due to increased cannibalization rates) for first-time C57Bl/6 J mouse mothers, with high rates of offspring death also observed for dams fed the low fat diet (following the first pregnancy).

Due to the low survival rates of litters born to dams fed a high fat diet, we were unable to compare the effects of maternal consumption of the high or low fat diet on offspring outcomes in a representative pup of each sex from each litter (with litter as *n* = 1/sex), as recommended by some statisticians [33]. As litter sizes were not significantly different between treatments, it was unlikely that in utero death contributed towards the increased size of pups born to dams fed a high fat diet. Our findings of reduced sex ratio (fewer males than females) born to dams fed a high fat diet are consistent with the *Sex Allocation Hypothesis* of Trivers and Wallard (1973), who state that; “as maternal condition declines, the adult female tends to produce a lower ratio of males to females” [54]. These negative effects of maternal high fat diet on offspring survival (and sex ratio) are important limitations for consideration in this study, and substantially reduced our statistical power for measures done in offspring.

We also recognize the limitations of using rodents as model animals to examine lung development. In rodents, lung alveolarisation is completed post-natally, unlike humans and sheep [38]. However, we report findings that are consistent with those of other researchers who have used mice, rats, and sheep as model animals to investigate the effects of maternal high fat diet consumption (or overnutrition) on offspring lung development. Similarly, our findings largely recapitulate the clinical phenotypes of obese asthma in children and adults. Our model could be useful to measure the potential interacting or exacerbating effects of maternal atopy or respiratory infection during pregnancy on the obese asthma phenotype in childhood. Similarly, the influences of other environmental exposures during pregnancy and early life will be important to explore, including; allergen exposure, pollution, living with pets, sun exposure, activity and other diet influences as well as the importance of the timing of high fat diet exposure, and the influence of high fat diet through the gut and/or lung microbiomes.

Our findings of increased body weight in offspring, were very similar to those of other research teams, who fed C57Bl/6 J dams a high fat diet for 3 months prior to mating, with offspring exhibiting increased body weights at 2 weeks of age, and preference for sugary and fatty foods [55]. Similar findings on the effects of maternal high fat diet on 2 week-old offspring body weight have also been reported, when C57Bl/6 J dams were fed a high fat diet for a shorter period of time (for ≤8 weeks pre-mating) [56, 57]. The effects of maternal high fat diet on body weight of young C57Bl/6 J offspring coincide with other signs of adiposity, including body composition and adipose tissue weights. Krasnow et al (2011) described that newborn (P0) pup born to dams fed a high fat diet for 4 weeks before mating had increased body weights, percentage body fat and hepatic fat levels [51]. Maternal high fat diet for 6 weeks prior to mating, increased body weight, WAT deposit weights and blood triglyceride levels in 3 week-old offspring [58]. Consumption of a high fat diet during gestation and lactation increased body weights, WAT deposit weights and blood triglyceride, glucose and insulin levels in 3 week-old offspring [57]. Dinger et al (2016) reported that maternal high fat diet during lactation increased body weight and epigonadal WAT deposits in 3 week-old offspring [40]. The effects of maternal high fat diet in promoting increased somatic growth of offspring, including body length, have been previously reported as epigenetically heritable features (across multiple generations) linked with changes in growth hormone signaling [59]. While we observed increases in both body weight and length, this was accompanied by increases in BMI, suggesting the changes in weight exceeded those effects on length. Together, these data suggest that maternal high fat diet consumption increases multiple signs of obesity in young C57Bl/6 J offspring.

## Conclusion

In conclusion, we report here that maternal intake of a high fat diet and the establishment of metabolic dysfunction prior to pregnancy may modify the development of the lungs of offspring, by increasing body size, lung volume and potentially causing changes in lung morphology, which need to verified through further experimentation. These effects may be unrelated to inflammation in offspring, but could be influenced by in utero exposure to maternal inflammation induced by ongoing consumption of the high fat diet. Our model may recapitulate clinical phenotypes of pediatric and adult obese asthma in humans, and can be used in future studies to explore the influence of environmental exposures on the development of metabolic and respiratory dysfunction in early life, to potentially identify new interventions that promote better metabolic health during pregnancy.

## Abbreviations

(S)G: (Specific) tissue damping
(S)H: (Specific) tissue elastance
(S)R_aw_: (Specific) airway resistance
BALF: Bronchoalveolar lavage fluid
BMI: Body mass index
FOT: Forced oscillation technique
GTT: Glucose tolerance test
I_aw_: Inertance
IL-: Interleukin-
PEEP: Positive end expiratory pressure
P_rs_: Transrespiratory pressure
R_n_: Newtonian airway resistance
TGV: Thoracic gas volume
TNF: Tumour necrosis factor
WAT: White adipose tissue
Z_rs_: Respiratory system impedance
η; G/H: Hysteresivity
