# Correction to: Maternal high fat diet compromises survival and modulates lung development of offspring, and impairs lung function of dams (female mice)

**DOI:** 10.1186/s12931-021-01681-4

**Published:** 2021-03-14

**Authors:** Jordan Smoothy, Alexander N. Larcombe, Emily K. Chivers, Vance B. Matthews, Shelley Gorman

**Affiliations:** 1 Telethon Kids Institute, University of Western Australia, Northern Entrance Perth Children’s Hospital, 15 Hospital Ave, Nedlands, WA 6009 Australia; 2 School of Biomedical Sciences, University of Western Australia, Perth, Australia; 3 School of Public Health, Curtin University, Perth, WA 6845 Australia

## Correction to: Respir Res (2019) 20:21 https://doi.org/10.1186/s12931-019-0976-3

Following publication of the original article [1], and upon recent review of the datasets of this manuscript, the authors have discovered some inconsistencies/inaccuracies in the number of animals per group reported in some figure legends (Figs. 3b, 5 and 6).

These are corrected as below:

- Fig. 3b: Fasting insulin levels at 9 (dams fed low fat diet, n = 14; or, high fat diet, n = 10) and 12 weeks (dams fed low fat diet, n = 12; or, high fat diet, n = 12).
- Fig. 5: Lung responsiveness to methacholine (dams fed low fat diet, n = 12; or, high fat diet, n = 11).
- Fig. 6: Serum levels of cytokines or adipokines in offspring born from first (dams fed low fat diet, n = 5–10; or, high fat diet, n = 5–10) or second (dams fed low fat diet, n = 10–12; or, high fat diet, n = 3–5) pregnancies.

The authors apologise for any inconvenience caused.
